# Clinical Trials of Stem Cell Therapy for Cerebral Ischemic Stroke

**DOI:** 10.3390/ijms21197380

**Published:** 2020-10-06

**Authors:** Masahito Kawabori, Hideo Shichinohe, Satoshi Kuroda, Kiyohiro Houkin

**Affiliations:** 1 Department of Neurosurgery, Hokkaido University Graduate School of Medicine, Sapporo 060-8638, Japan; hshichi@med.hokudai.ac.jp (H.S.); houkin@med.hokudai.ac.jp (K.H.); 2 Department of Neurosurgery, Toyama University, Toyama 930-0194, Japan; skuroda@med.u-toyama.ac.jp

**Keywords:** stem cell, regenerative medicine, ischemic stroke, clinical trials, transplantation

## Abstract

Despite recent developments in innovative treatment strategies, stroke remains one of the leading causes of death and disability worldwide. Stem cell therapy is currently attracting much attention due to its potential for exerting significant therapeutic effects on stroke patients. Various types of cells, including bone marrow mononuclear cells, bone marrow/adipose-derived stem/stromal cells, umbilical cord blood cells, neural stem cells, and olfactory ensheathing cells have enhanced neurological outcomes in animal stroke models. These stem cells have also been tested via clinical trials involving stroke patients. In this article, the authors review potential molecular mechanisms underlying neural recovery associated with stem cell treatment, as well as recent advances in stem cell therapy, with particular reference to clinical trials and future prospects for such therapy in treating stroke.

## 1. Introduction

**Figure 1:**
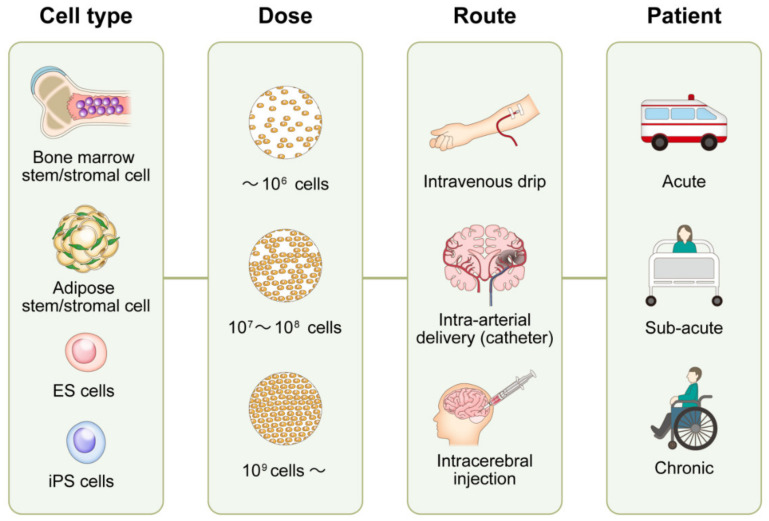
Unsolved issues regarding stem cell treatment for ischemic stroke. The most effective and safest method of stem cell therapy has not been established. The challenges include the choice of cell, cell dose, transplantation routes, and patient type. ES cell: embryonic stem cell, iPS: induced pluripotent stem cell.

Besides the rapidly expanding use of thrombectomy as a remedy for acute ischemic stroke [1], few drugs that can effectively recover its sequelae have been developed. Lately, stem cell therapy has been recognized as a promising strategy that functionally enhances recovery from ischemic stroke. Thus, a variety of cells, including bone marrow mononuclear cells (BMMNCs) [2,3,4,5,6,7,8,9,10,11,12,13,14,15,16,17,18,19,20,21], bone marrow/adipose-derived stem/stromal cells (BMSCs/ADMSCs) [4,6,7,22,23,24,25,26,27,28,29,30,31], umbilical cord blood cells (UCBCs) and hematopoietic stem cells [32,33,34,35,36,37], neural stem cells (NSCs) [34,36,38,39,40,41,42,43], olfactory ensheathing cells (OECs) [38], and fetal porcine cells [44], have been explored as candidate donors. Animal studies have indicated that such cells may ameliorate the neurological deficits that follow cerebral stroke, and some have been tested in clinical trials with somewhat favorable results. However, many issues, such as the need to develop techniques that maximally enhance the effects of cell therapy on stroke, remain unresolved, and require clarification [45,46,47,48]. These issues relate to optimal cell types, cell doses, transplantation routes, and candidate patient types (Figure 1). In addition to the refinement of scientific aspects, cell therapy requires an assessment from a commercial point of view, to be successfully distributed as a new therapeutic method. Cell therapy requires the implementation of good manufacturing practice (GMP) grade production method at a reasonable cost for production, preservation, and transfer of the cells. Here, the authors review potential mechanisms underlying stem cell-associated neural recovery, the current status of clinical trials, and future prospects for utilizing cell therapy against ischemic stroke.

## 2. Pathophysiology of Ischemic Stroke and Therapeutic Targets

To coordinate bodily processes, the brain requires approximately 20% of the entire cardiac output of glucose and oxygen, which is equivalent to only 2% of total body weight [49,50]. As the brain stores little or no energy on its own, disruption of the energy supply, even for a short duration, may lead to catastrophic damage. Ischemic stroke is often caused by the occlusion of a single blood vessel, which subsequently affects its downstream branches via deprivation of glucose and oxygen. Although brain arteries possess a network of collateral vessels that compensate by ensuring the delivery of blood, it is often insufficient to rescue the whole ischemic area, as a result of which ischemic areas closer to the occluded vessel become more susceptible to receiving less blood. Theoretically, an affected brain may be divided into two different damaged areas, namely the ischemic core and the penumbra. Because the blood flow in the ischemic core is lower than the threshold required for cell survival, its cells are irreversibly damaged and die due to necrosis, for which there is no rescue. In contrast, blood flow at the penumbra is too low to support neurological functions but provides the minimal energy required for preventing cells from immediate death, allowing the brain cells to recover if blood flow is restored in time [51]. Therefore, current treatment strategies for stem cell transplantation involve rescuing the penumbra before it dies, or regaining a new neuronal network via cell transplantation [52]. The ischemic cascade in the penumbra progresses with time. Events including the depletion of adenosine triphosphate (ATP); disturbance of ionic concentrations of sodium, potassium, and calcium, increased lactate, acidosis, accumulation of oxygen free radicals, the release of excitotoxic glutamate, and intracellular accumulation of water, may be initiated within minutes to hours following the onset (acute phase) of stroke. This acute phase may be followed by events such as apoptosis of neuronal cells, infiltration, and activation of inflammatory cells (neutrophil, monocyte, and microglia), vasogenic edema, and increase in intracranial pressure, within hours to weeks (subacute phase) [53]. Although the condition of the brain appears to stabilize during the chronic phase (months to years), recent findings indicate that inflammation and blood–brain barrier leakage, which are detrimental to brain recovery, may continue [54,55,56,57].

Currently, standardized treatments, such as thrombectomy and recombinant tissue plasminogen activator (r-tPA) therapy, are applied during the acute phase (<4.5 h). These treatments aim to recanalize the occluded vessel and rescue the penumbra. However, it is often difficult to successfully apply these treatments during short time periods, and reportedly, only 5–10% of all stroke patients become eligible for such treatment [58]. Stem cell therapy, which is known to be effective in all three phases, namely acute, subacute, and chronic, reportedly exerts multiple effects on animal models, such as extending the penumbra period (acute phase), inhibiting unwarranted inflammation (subacute phase), and initiating neuro/angiogenesis (chronic phase) [59].

## 3. Potential Mechanisms of Stem Cell Therapy

Extensive efforts have been made to elucidate the mode of action underlying the treatment of ischemic stroke via stem cell transplantation, resulting in the publication of multiple descriptive reviews [60,61,62,63,64]. Briefly, transplanted cells are known to exert a variety of neuro- and vascular-protective effects during the various phases of an ischemic stroke. The transplanted cells not only reorganize the neuronal network but also reduce local and systemic inflammation, support axonal regeneration and synaptic sprouting, and reduce glial scars. These mechanisms can be sub-categorized into two distinct types: (i) cell differentiation (cell replacement); (ii) secretion of paracrine factors (Bystander effect).

### 3.1. Cell Differentiation

Cell replacement may be achieved via the differentiation of transplanted cells into neuronal or vascular cells, which compensates for lost functions, or via the direct settlement and development of neuronal progenitor cells [65,66]. Azizi et al. (1998) examined, ex vivo expanded bone marrow-derived stem cell settlement in the ischemic brain, and indicated that 20% of human BMSCs transplanted into a rat brain remained alive 72 d after infusion, and showed neuronal phenotypes [67]. Our group demonstrated that in vitro chemical induction of BMSCs reduced the expression of mesenchymal cell lineage genes and enhanced the expression of neural genes associated with the release of trophic factors [68,69]. An in vivo study revealed that approximately 50% of engrafted stem cells in the ischemic brain expressed a neuronal phenotype 2 months following cell transplantation [70,71,72]. The migration of stem cells to the damaged area is also reported [72]. Intracerebrally injected stem cells express the CXCR4 receptor, which can bind to stromal cell-derived factor-1 (SDF-1), a chemoattractant. SDF-1 is expressed from the damaged brain and the stem cell uses this CXCR4/SDF-1 axis to migrate to the damaged regions of the brain. However, whether these transplanted and phenotype-altered cells actually compensate for the lost neurological network remains unclear [35].

### 3.2. Bystander Effect of Stem Cells

The secretion of paracrine factors is an important aspect of the functional multipotency of stem cells, wherein these cells secrete various trophic factors such as cytokines, chemokines, and exosomes, which ameliorate neuronal damage or regenerate new neuronal circuits [73,74,75]. In addition to promoting anti-inflammatory and immunomodulatory effects, these factors induce anti-apoptotic effects and mobilize endogenous stem cells (NSC))/neural progenitor cells (NPCs) [76]. These factors are released into the surrounding environment via direct permeation or extracellular vesicles (EV), and directly ameliorate ischemic damage and down-regulate local as well as systemic inflammation via peripheral immune organs, such as the spleen and the thymus [77,78]. EVs are membrane structures of lipid bilayer nanoparticles that transport proteins, lipids, and nucleic acids through endocytosis. EVs are attracting attention due to their low immunogenicity and high blood-brain barrier (BBB) permeability, which reduces damage and facilitates recovery. These properties along with its versatility make EVs promising as vehicles for drug delivery [79]. Recent reports suggest that EVs can ameliorate ischemic damage through multiple mechanisms including upregulation of angiogenesis, neurogenesis, and modulation of autophagy after ischemic stroke [80,81]. Besides rescuing damaged brain cells, these factors accelerate the regeneration of in-house stem cells. Trophic factors fuel the proliferation of host neuronal progenitor cells, especially of those located around the subventricular zone (SVZ), which are normally inactive.

## 4. Key Aspects of Clinical Trials

### 4.1. Overview of Clinical Trial Results

A comprehensive search of the clincaltrials.gov database was performed using the search criteria “ischemic stroke” and “stem cell” on 23/04/2020. A total of 52 results were returned, and the status “completed” for the trial were then manually screened for its publication, following which PubMed articles linked to clincaltrials.gov were evaluated for additional information where appropriate. Further PubMed searches were performed using the terms “ischemic stroke*” and “stem cell” or “neural stem cell (or NSC),” “mesenchymal stem cell or mesenchymal stromal cell” (or “MSC”), “mononuclear cell or mononuclear precursor cell” (or “MNC”) and “Schwann cell” (or “SC”), “olfactory ensheathing or olfactory glia (or OEC)” or “oligodendrocyte precursor (or OPC).” Each article type was then restricted to “clinical trial” to identify any other published studies that had not been registered on clinicaltrials.gov. Additional searches were performed to identify case studies where appropriate. Cell type, cell source, dose, route, timing, patient number, assessment modality, and major outcome were extracted from the manuscript. Cell doses were re-calculated at 60 kg for each patient if the dose was only stated in terms of the number of cells per kilogram (cells/kg). A total of 43 published clinical trials were obtained (Table 1). The trials were categorized into acute (treatment within a week from stroke onset), sub-acute (treatment between 1 week and 6 months from onset), and chronic (treatment after 6 months from onset). Some trials contained multiple treatment time points and were divided by the actual timing of treatment listed in the manuscript.

**Figure 2:**
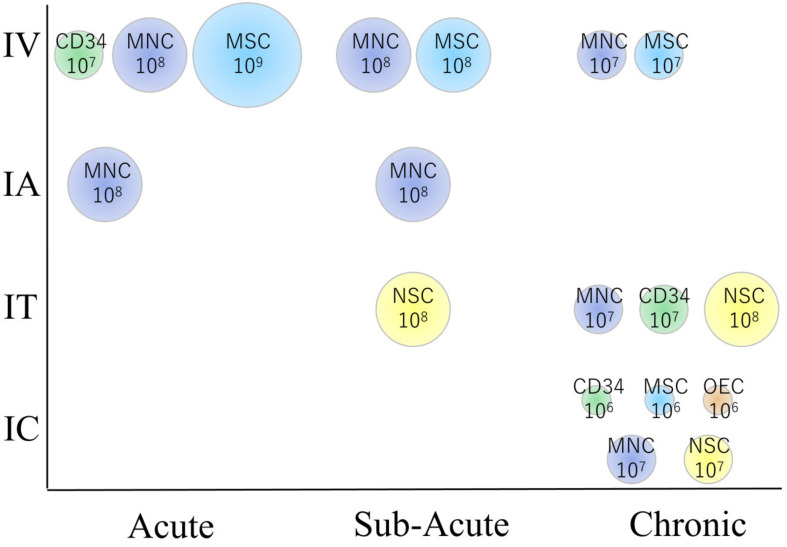
The relationship between cell types, dose, and patient characteristics in clinical trials. Note that intravenous transplantation is preferred in the acute phase, while intracerebral transplantation is preferred in the chronic phase. MNC: CD34: CD34 positive hematopoietic stem cells derived from mononuclear cells, MSC: Mesenchymal stem/stromal cell, NSC: Neural stem/progenitor cell, OEC: Olfactory ensheathing cell. The number represents the approximate amount of cells transplanted per patient (cells/body).

The methodologies differed widely between trials as well as countries that the trials were executed in (Table 1). Autologous BMMNCs account for the largest portion of cells, followed by autologous bone marrow stem/stromal cells. Small amounts of other sources of cells, such as UCBCs and adipose-derived stem/stromal cells, or neuronal progenitor cells, are also used. Cell doses, which differed widely, ranged between 1 × 10^6^ to 1 × 10^9^, while transplantation routes consisted of intravenous (IV), intraarterial (IA), intrathecal or intracerebroventricular (IT), and intracerebral (IC) routes. Intravascular routes (IV and IA) appear to offer higher cell numbers (up to 10^9^ cells) than those of IC transplantation (10^6-7^) (Figure 2). This is because IC transplantation limits the amount of cells that can be transplanted to avoid a mass effect on the brain, whereas intravascular transplantation does not. IV transplantation appears to be preferable in the acute to sub-acute phase, while IC or IT transplantation is mostly performed during the chronic phase. A majority of these trials were of a preliminary nature and control groups were not set up, whereas some trials did set up control groups consisting of unblinded or blinded patients. All but one study reported no detrimental effects due to cell therapy, while the single study that did, used xenogeneic fetal porcine cells and reported that cell transplantation exerted a negative effect causing seizures and motor function aggravation, which led to the termination of the trial. Although assessment modalities also differed widely between trials, modified Rankin Scale (mRS), National Institute of Health Stroke Scale (NIHSS), and Barthel index were commonly applied.

#### 4.1.1. The Acute Phase of Stroke

Cell transplantation within a week from the onset of a stroke is defined in this review as treatment during the acute phase. Because it is difficult to expand autologous mesenchymal stem cells under ex-vivo conditions within this time frame, BMMNCs or allogenic cells were selected [9,10,12,16,25,32,35,36]. The trials used IV or IA transplantation and did not use IC injections. Spontaneous recovery strongly influenced the final result within this time frame, and thus should be taken into consideration when assessing results. While the smaller, early phase studies that did not set up control groups reported good clinical recovery, two studies of IV and IA transplantation that used control patients reported an absence of statistical difference between functional recovery and control groups [12,25]. However, referring to their post-hoc analysis, one study declared that patients who received cells between 24 and 36 h (trial inclusion 24–48 h) showed a significant improvement in motor recovery one year following treatment [25]. This indicated that patients receiving their BMSCs early via IV injection benefited from the treatment. Currently, studies using these cells under new time course (24–36 h) conditions are ongoing in Japan [82].

#### 4.1.2. The Sub-Acute Phase of Stroke

Cell transplantation after a week for up to 6 months is considered to be sub-acute treatment [2,3,5,8,11,13,14,15,17,20,22,24,26,27,28,34,36,42]. In addition to BMMNCs, autologous BMSCs are used for ex-vivo expansion within a time frame of approximately 1 month. IV or IA transplantation accounts for the majority of trials, while IT transplantation has also been reported. The results of these studies are mostly similar to the favorable results reported in the acute phase of trials using small samples, while larger randomized trials showed heterogeneous efficacy [8,11,14,17,22,24,27,28]. A large trial performed by Prasad et al. included 118 patients, half of which received approximately 3 × 10^7^ autologous BMMNCs between 7 and 10 d following the insult [14]. This phase II multicenter, parallel-group, randomized accessor blinded trial revealed that, although IV infusion of BMMNCs was safe, it did not exert any beneficial effects (BI) on stroke outcome. Lee et al. reported that IV injection of 1 × 10^8^ BMSCs resulted in better recovery and reduced mortality for up to 5 years from treatment initiation, compared with randomized controls [28], whereas Jaillard et al. did not report an overall benefit [27]. Differences in cell processing procedures, patient types, and timing make arriving at a specific conclusion much more difficult. The results of IA treatments also differed between trials. Bhatia reported a good trend (*P* = 0.06) of recovery via IA transplantation of autologous BMMNCs [8], while others did not report a difference [11,17]. A recent report by Savitz et al. discussed a new aspect regarding logistics [17]. Autologous stem cells are mainly processed at the transplantation site and do not require cell preservation while transferring. However, it is impossible to make these commercially available unless a cell preservation and logistics process is developed for wide commercial distribution. They reported that bone marrow extracted from the patient was transferred to a sorting facility, and shipped back to the hospital for transplantation. These procedures are considered very important for cell transplantation purposes especially when using autologous stem cells.

#### 4.1.3. The Chronic Phase of Stroke

Initiating treatment 6 months after an ischemic stroke is considered as treatment during the chronic phase [4,5,6,7,18,19,23,29,30,31,33,36,37,38,39,40,41,42,43,44,66]. Currently, no effective treatments are available for this phase, and thus the establishment of an effective treatment process is highly anticipated. Interestingly, IC or IT injections account for most transplantation routes within this time frame. However, only one study investigating IC transplantation had used control patients, and this study reported that IC transplantation of CD34 positive hematopoietic cells initiated marked neurological recovery compared with that of the control [38]. Although the number of patients screened was small (*N* = 6 each), IT injection resulted in better recovery compared with that of the control [42]. The results of IV transplantation varied between trials, where some studies reported significant recovery compared with that of the control [5,7], while others did not [4,6,21]. Randomized clinical trials using larger patient samples are currently ongoing (NCT02448641, NCT02448641) and the results are expected soon.

## 5. Unsolved Issues Associated with Optimal Treatment

### 5.1. Stem Cell Types

Many stem cell types, including mononuclear cells (MNCs), MSCs, OECs, and NSCs have been intensively examined as promising sources and tested via clinical trials, as previously mentioned. Some cells use gene-modification processes to enhance the release of trophic factors and survival [30,31]. Autologous cells (MNCs, MSCs, OECs) possess the advantage of being associated with a low risk for post-transplant rejection and allergies, whereas allogenic cells (MSCs, NSCs) are considered to be advantageous due to easier accessibility resulting from large-scale manufacturing and availability of standardized stocks. Clinical trials discussed here show a trend of moving from autologous to allogeneic cells, which aims for large-scale manufacturing for commercial purposes. Prior to distributing an available cell source for commercial purposes, several factors such as safety, efficiency, cost, and feasibility of manufacturing on a large scale, must be taken into consideration. Recent reports indicate that MSCs from the same bone marrow may express different functional and molecular phenotypes if produced using different facilities and methods [83]. This is indicative of the difficulties encountered in maintaining consistent quality during cell preparation. Several basic studies have compared the efficacy of different cell sources as treatments [84,85]. However, each stem cell type comes with its own benefits and drawbacks, and at present, which cell type represents the most beneficial treatment remains unclear.

#### 5.1.1. MNCs

An advantage associated with MNCs is that these may be obtained from patients without resorting to ex-vivo expansion. Approximately, 1 × 10^8^ MNCs can be obtained from 50 mL of bone marrow, and transplanted immediately following isolation [83]. Therefore, this cell type is widely used in the acute and subacute phases, that nearly half of the trials used bone marrow-derived mononuclear cells (Table 1). However, a disadvantage associated with using MNCs is that MNCs only contain very small amounts of MSCs (0.1–0.01% of MNCs), which, according to some researchers, casts doubts regarding its efficacy.

#### 5.1.2. Hematopoietic Stem Cells (CD34 Positive)

Hematopoietic stem cells, expressing CD34, which are obtained from both bone marrow and peripheral blood are also frequently used. These cells, which have a long history of being harvested and used to treat hematological disorders under clinical conditions, are considered safe for clinical use. These cells show a strong capacity for angiogenesis, as witnessed in diseases such as myocardial infarction and limb ischemia [86,87,88], and show potential for reorganizing the vascular network in the brain [89]. An advantage of using these cells is that ex-vivo cell expansion, which requires time and effort, is not required. However, these cells show limited capacity for neuronal differentiation and are thus unable to complete the complex restoration process needed to repair ischemic stroke-related damage. These cells tend to accumulate during inflammation, and may not reach the brain when other organs, such as heart and lung, are inflamed [90].

#### 5.1.3. MSCs

The nomenclature of MSCs (stromal or stem cells) is convoluted. The International Society for Cell & Gene Therapy (ISCT) Mesenchymal Stromal Cell Committee has established the minimal criteria that are required for a cell to qualify as a mesenchymal stromal cell: (i) plastic-adherence; (ii) CD73, CD90, and CD105 expression; 3) absence of expression of hematopoietic and endothelial markers CD11b, CD14, CD19, CD34, CD45, CD79a, and HLA-DR; (iv) capable of in vitro differentiation into adipocyte, chondrocyte, and osteoblast lineages [91,92]. However, it was later observed that some cell-surface markers displayed an ability to be reversibly upregulated or downregulated according to cell culture conditions [93,94,95]. The use of “stromal” and “stem” to describe MSCs is almost equivalently found in the literature, and the ISCT suggests that “mesenchymal stromal cell” should be used to describe bulk unfractionated populations, which include fibroblasts, myofibroblasts, and stem/progenitor cells, whereas “mesenchymal stem cell” should be used for purified stem/progenitor cells [96]. An abundance of preclinical evidence indicates that MSCs possess an ability to ameliorate tissue damage and facilitate functional recovery via multiple processes, including immunomodulation, pro-angiogenic signaling, neurotrophic factor secretion, and neural differentiation [70,71,97,98]. MSCs can be harvested from bone marrow, abdominal fat tissue, teeth, umbilical cord blood, and Wharton’s Jelly. MSCs have several advantages over other stem cells due to well-established harvesting methods, low risk for tumorigenicity, and the absence of ethical issues [64]. MSCs possess a unique immune tolerance, where even allogenic MSCs, which do not show immunological rejection responses, are approved for graft vs host disease (GvHD) treatment in many countries [99]. Gene modification of BMSCs has also been reported. SanBio developed SB623 cells through transient transfection of a plasmid containing the human Notch-1 intracellular domain [100]. This cell showed better neuroprotective properties, via higher trophic factor secretion, stronger anti-inflammatory effect, and neuro-/angiogenesis. They recently reported that SB623 was associated with a rate of recovery from chronic traumatic brain injury, which was statistically significant (unpublished data).

#### 5.1.4. NSCs

NSCs are multipotent progenitor cells capable of integrating with the host brain by transforming into neural cells, oligodendrocytes, and astrocytes [101,102]. These cells survive in the host brain and exhibit neuroprotective effects through extending processes, expressing neurotransmitters, and forming functional synapses [103,104]. Although these cells are mostly found during the development of the fetal CNS, they are also present in a limited number of other regions of the adult brain, such as the subventricular zone next to the cerebral lateral ventricle [105]. Although NSCs appear to be ideal for refilling lost neuronal networks, the cells need to be harvested from the fetus, which poses ethical issues and there is the possibility of immune rejection by the host. Other potential concerns include whether NSCs can initiate angiogenesis, differentiate to vascular structures, since brain reconstruction requires other cell types including vascular cells, such as endothelial cells, and remain pluripotent after adulthood.

#### 5.1.5. OECs

OECs surround olfactory neurons, and function as scavengers of pathogens and debris around the border between the CNS and the nasal mucosa. Additionally, they reportedly express neurotrophic factors that facilitate olfactory regeneration. OECs can be harvested from the nasal mucosa and the olfactory bulb. These cells secrete neurotrophic factors, such as the stromal cell-derived factor 1-a (SDF-1 a), and the brain-derived neurotrophic factor (BDNF), which promote neuronal regeneration [106,107]. These cells have been extensively examined in relation to spinal cord injury, but investigating its usefulness in treating ischemic stroke has just started [108,109,110,111]. Data indicating its potential or detrimental nature are scant.

#### 5.1.6. Other Cell Types

Embryonic stem (ES) cells and induced pluripotent stem (iPS) cells have also been examined in preclinical studies [112,113,114]. Their pluripotency is an attractive characteristic in relation to its usefulness in treatment. However, data regarding clinical trials of these cells are currently unavailable due to factors associated with ethics and tumorigenicity.

### 5.2. Cell Dose and Route

IV transplantation has the advantage of showing the lowest invasiveness, thereby allowing multiple injections. The method also does not require special equipment for transplantation. However, despite its efficacy, small amounts of cells are often found in the damaged lesion, and most cells are trapped in the lungs [71]. The bystander effect exerted by neurotrophic factors resulting in the amelioration of apoptosis and inflammation are considered as the main therapeutic mechanisms underlying IV transplantation. This is useful in the acute phase of ischemic stroke, but may not be beneficial in the chronic phase during which cell damage and inflammation are mostly settled. The IA approach is considered superior to IV administering in delivering more cells to the lesion. However, recent reports have indicated that this method is not effective for cell engraftment in the brain [2,3,15]. Additional ischemic damage caused by cell clusters clogging the arteries is a drawback of this method [17]. IT application, which can deliver a large number of cells to the subarachnoid space, is less invasive relative to IC transplantation. However, the rate of cell engraftment is unclear, and complications, such as hydrocephalus and liquorrhea, may arise. The IC approach of directly administering cells achieves the highest level of cell engraftment but requires invasive surgery, and the risk of additional brain damage being caused by injection needles should not be underestimated [115].

As previously mentioned, IV and IA transplantations, using a large number of cells ranging up to 10e9 cells, are preferred in the acute and sub-acute phases, whereas, in the chronic phase, IC transplantation with a smaller cell dose of 10^7^ cells is preferred. Interestingly, most IV/IA transplanted stem cells are not found in the brain but the other organs, such as lungs, spleen, and bladder [2,3,9,15]. Rosado-de-Castro et al. transplanted technetium-99m labeled BMMNCs intravenously and intra-arterially into sub-acute stroke patients, and found that only 0.6–0.9 % of cells were present in the brain 2–24 h after transplantation [15]. They reported that the IA transplantation group had higher radioactive counts in the liver (2 h: 40% and 24 h: 47%) and spleen (2 h: 6% and 24 h: 7%), and low counts in the lungs (2 h: 7% and 24 h: 4%), compared to IV transplantation (liver 14% and 19%, spleen 2% and 3%, and lung 21% and 7%, respectively). According to this result, intravenously and intra-arterially transplanted cells are distributed differently soon after transplantation, following which intravenously transplanted cells are found in the lung, while intra-arterially transplanted cells are in the liver. This result is similar to other reports that intra-arterially transplanted technetium-99m labeled BMMNCs, which were found in the brain 2 h after ischemia with the main uptake occurring in the liver, lungs, spleen, kidneys, and bladder. After 24 h, the cells were hardly distinguishable in the brain, while uptake was still observed in the other organs [2]. These results indicate that cells that are transplanted intravenously or intra-arterially are unable to stay in the brain for a long time. We have recently revealed that intracerebrally injected iron labeled BMSCs can migrate, settle in the ischemic area, and survive for more than 2 years (unpublished data) [116].

### 5.3. Patient Characteristics and Outcome Measure

It is difficult to estimate which pathological aspect (timing, stroke type, comorbidity disease) of a patient will most benefit from stem cell treatment, via the use of animal models. Clinical trials involving a large number of patients or real-world data are required to resolve this issue. Outcome evaluation needs to be adequately refined to accurately monitor the results of clinical trials. mRS, NIHSS, and BI are often used in clinical trials, but mRS is too broad-based to detect small differences, while NIHSS is mostly intended for acute assessment of patients.

## 6. Future Directions

While the results of the clinical trials are promising, there are other factors such as regulatory approval and the overall cost to be considered for the widespread use of stem cells in the treatment of ischemic stroke. The key is to achieve a balance between the quality of cells produced and the costs involved, two apparently conflicting parameters.

### 6.1. Producing Good Cells (GMP Grade)

Most of the clinical trials, especially those using autologous cells, were performed completely within a single hospital, where cell preparation was also done on-site. Some clinical trials mentioned about the GMP grade cell production, while the others did not. GMP is a system for ensuring quality controlled drug production to minimize the risks. It covers all aspects of production such as the handling and checking of materials, producing drugs according to the standard operating procedures (SOP), appropriate packing of drugs, and delivery management. Following GMP is time-consuming and costly, however, the cells will not qualify for drug use in many countries unless GMP is followed. The problem with this is that each country possesses its own GMP requirement. Pharmaceutical companies need to fulfill the requirement of the country where drug production will be carried out. Many regulatory agencies are working together to develop a set of common rules for drug approval, and this policy can help achieve faster drug development and approval in the future.

### 6.2. Producing Cells at Low Cost

Many clinical trials are executed as an investigator-oriented trial, where government or public funds are used for cell production. Cell preparation can be very expensive and to normalize stem cells as a standard treatment method, the cost needs to be minimized. Current cell expanding procedures requires the expertise of experienced technicians. Allogeneic stem cells are suitable for bulk production using automated cell producing machines; however, there is a need for innovative technology when it comes to autologous stem cells, which are made-to-order and are difficult to be adapted for automated production. Cell logistics are another key issue. Stem cells differ from ordinary low-molecular drug compounds in that stem cell efficacy is dependent on viability, which means that adequate cell preservation is mandatory. Cryopreservation and shipping of stem cells are often adopted in the clinical trials, but the reagent for cryopreservation contains DMSO, a possible toxic reagent, and shipping under extremely low temperature (using liquid nitrogen) is costly. Therefore, the production and transfer of stem cells at an affordable cost require further optimization.

## 7. Conclusions

Stem cell therapy is expected to ameliorate the sequelae of those ischemic stroke patients who have reached the acute phase, a stage at which no proven treatment is currently available. The results of clinical trials are promising, in the sense that most methods used for stem cell transplantation appear to be safe. It seems that intravenous or intra-arterial transplantation is preferred in the acute phase, where the aim is to ameliorate systemic and local inflammation and cell engraftment is not required. Alternatively, intracerebral transplantation is preferred in the chronic phase, where cell engraftment is considered the objective of cell therapy. However, optimal parameters including the choice of cell type, cell dose, and patient characteristics remain elusive and further research is needed for maximizing the effects of the proposed methods. To achieve this, it is expected that the integration of pre-clinical and clinical research will take place in the near future.

## Figures and Tables

**Table 1:** Published clinical trials using stem cells for ischemic stroke.

Reference Number	Country	Cell Type	Cell Source	Dose	Route	Transplant Timing	Treated Patient Number (Control)	Assessment Modality	Major Outcome
Acute									
[16]	USA	Autologous	BMMNC	4–6 × 10^8^	IV	1–3 D	10	BI, mRS, NIHSS	showed good neurological recovery
[25]	USA	Allogeneic	BMSC	1.2 × 10^9^	IV	1–2 D	65 (58)	mRS, NIHSS, BI	No difference for neurological recovery (primary endpoint), but earlier timing (24-36 h) may be beneficial
[35]	USA	Allogeneic	UCBC	1.2 × 10^6^ (CD34+)	IV	3–9 D	10	mRS, NIHSS	Safe
[10]	Brazil	Autologous	BMMNC	5–60 × 10^7^	IA	3–10 D	20	mRS, NIHSS	30% of the patients showed satisfactory clinical outcome
[12]	Spain	Autologous	BMMNC	1.6 × 10^8^	IA	5–9 D	10(10)	mRS, BI, NIHSS	No difference in neurological function
[9]	Brazil	Autologous	BMMNC	3 × 10^7^	IA	9 D	1	SPECT	Brain/liver/spleen uptake at 8 h
[32]	UK	Autologous	CD34+ (BM)	1–3 × 10^6^	IA	1 W	5	mRS, NIHSS	Good recovery was observed
[36]	China	Allogeneic	UCBC & NPC	3 × 10^7^ (UC: IV), 1.5 × 10^7^ (UC: IT), 1.8 × 10^7^ (NPC: IT)	IV &IT	1 W	1	NIHSS, BI, mRS	Showed some degree of neurological recovery
**Sub-Acute**									
[13]	India	Autologous	BMMNC	2–19 × 10^8^	IV	2–4 W	11	NIHSS, BI, mRS, PET	Favorable outcomes were mostly found in early treatment group
[5]	India	Autologous	BMMNC	5 × 10^7^	IV	3–4 M	1(3)	FM, mBI	Safe
[15]	Brazil	Autologous	BMMNC	2–5 × 10^8^	IV	1–3 M	5	NIHSS	Cells in brain were scarce (1%), IV (21%) showed high cell distribution in lung compared with IV (7%)
[14]	India	Autologous	BMMNC	2.8 × 10e7	IV	18 D	59(59)	BI, mRS, NIHSS, PET	No significant recovery compared with control
[20]	Japan	Autologous	BMMNC	2.5–3.4 × 10^8^	IV	7–10 D	12	mRS, NIHSS, SPECT, PET	Better NIHSS (but not mRS, BI) recovery compared with historical control
[22]	Korea	Autologous	BMSC	1 × 10^8^	IV	1–2 M	5 (25)	BI, mRS, NIHSS	Cell treatment group showed better neurological recovery than control
[28]	Korea	Autologous	BMSC	1 × 10^8^	IV	2 M	16(36)	mRS, Survival	Better recovery, less mortality for 5 years
[26]	Japan	Autologous	BMSC	0.8–1.5 × 10^8^	IV	1–4 M	12	NIHSS	Recoveries were mainly seen 0–1 W from transplantation
[24]	China	Autologous	BMSC	3 × 10^8^	IV	1 M	12 (6)	mRS, NIHSS, BI	No neurological difference compared with control
[27]	France	Autologous	BMSC	1 or 3 × 10^8^	IV	1–2 M	16(15)	NIHSS, mRS, BI	No overall change, but motor functional evaluations indicated improvement
[36]	China	Allogeneic	UCBC & NPC	1.2 × 10^8^ (UC)	IV	2 & 3 M	2	NIHSS, BI, mRS	Showed some degree of neurological recovery
[2]	Brazil	Autologous	BMMNC	1–5 × 10^8^	IA	2–3 M	6	SPECT	Cells were found in the brain after 2 h, but not after 24 h
[3]	Brazil	Autologous	BMMNC	1–5 × 10^8^	IA	2–3 M	6	NIHSS, SPECT	Safe, but cells could not be seen 24 h after injection in 4 out of 6 patients
[15]	Brazil	Autologous	BMMNC	1-5 x 10^8^	IA	1–3 M	7	NIHSS	Cells in brain were scarce (1%), IA (41%) showed high cell distribution in liver compared with IV (13%)
[11]	Egypt	Autologous	BMMNC	1 × 10^6^	IA	2–4 W	21(18)	NIHSS, mRS, BI,	IA treatment did not improve neurological recovery compare with control
[8]	India	Autologous	BMMNC	5 × 10^8^	IA	1–2 W	10 (10)	BI, NIHSS, mRS	Good recovery was observed in treatment group (*P* = 0.06)
[17]	USA	Autologous	BMMNC (ALD)	3 × 10^6^	IA	2–3 W	29 (17)	mRS, NIHSS, BI	No statistical difference compared to control
[34]	China	Allogeneic	UCBC & NPC	2 × 10^7^	IA	11–22 D	3	mRS	Showed neurological recovery in 2 out of 3 patients
[42]	Russia	Allogeneic	Fetus neuronal cell	2 × 10^8^	IT	4 M	1	Karnovskii score	Cell treatment showed 33% increase in Score
[36]	China	Allogeneic	UCBC & NPC	3 × 10^7^ (UC: IV), 1.5 × 10^7^ (UC: IT), 1.8 × 10^7^ (NPC: IT)	IV & IT	2 W	1	NIHSS, BI, mRS	Showed some degree of neurological recovery
**Chronic**									
	India	Autologous	BMMNC	6–7 × 10^7^	IV	5–14 M	20(20)	FM, mBI, Ashworth	No difference compared with control
[21]	India	Autologous	BMMNC	5 × 10^7^	IV	6–15 M	11(9)	FM, mBI	Significant improvement in mBI, but not in FM
[4,5]	India	Autologous	BMSC	5–6 × 10^7^	IV	8–12 M	6(6)	BI, FM, Ashworth	No significant difference compared with control up to 4 years
[29]	USA	Allogeneic	BMSC (hypoxia treated)	1 × 10^8^	IV	7 M-25 Y	36	NIHSS, BI	Significant recovery was observed compared with baseline
[7]	India	Autologous	BMSC/BMMNC	5-6 × 10^7^	IV	3 M-2 Y	20(20)	FM, mBI	mBI showed significant improvement
[18]	India	Autologous	BMMNC	6 × 10^7^	IT	4 M-12 Y	14	FIM	Showed recovery, but this study included hemorrhagic stroke
[37]	China	Autologous	CD34+ (peripheral)	1–3 × 10^7^	IT	1–7 Y	8	NIHSS, BI	Patients showed recovery, but this may have been due to natural history
[42]	Russia	Allogeneic	Fetus neuronal cell	2 × 10^8^	IT	8 M-1.5 Y	6 (6)	Karnovskii score	Cell treatment groups showed better recovery
[23]	USA	Autologous	ADSC (no culture)	N.D.	IT (ICV)	1 Y	1	N.D.	Stable
[19]	Cuba	Autologous	BMMNC	1–5 × 10^7^	IC	3–5 Y	3	BI, NIHSS, SSS	Recovery compared with pre-operation was found
[33]	Taiwan	Autologous	CD34+ (peripheral)	3–8 × 10^6^	IC	6 M-5 Y	15(15)	NIHSS, ESS, mRS	Statistically significant recovery
[30,31]	USA	Allogeneic	BMSC (Gene modified)	2.5, 5, 10 × 10^6^	IC	7–36 M	18	ESS, NIHSS, FM	Neurological recovery (ESS, NIHSS, F-M test) was observed up to 2 years
[41]	USA	Allogeneic	Fetus neuronal cell	2 × 10^6^ (*n* = 8) or 6 × 10^6^ (*n* = 4)	IC	7 M-5 Y	12	BI, ESS, NIHSS	6 x 10^6^ showed better recovery than 2 x 10^6^
[39]	UK	Allogeneic	Fetus neuronal cell	2, 5, 10, 20 × 10^6^	IC	1–4 Y	11	NIHSS, BI, Ashworth	Neurological recovery (median NIHSS of 2) was observed
[43]	UK	Allogeneic	Fetus neuronal cell	2 × 10^7^	IC	2M-1 Y	23	ARAT	Upper limb function recovered from baseline
[40]	USA	Allogeneic	Fetus neuronal cell	5, 10 × 10^6^	IC	1–6 Y	18(4)	ESS, NIHSS, FM, ARAT	No difference for neurological recovery (primary endpoint), but showed partial recovery in some tests
[38]	China	Allogeneic	OEC	1 × 10^6^	IC	3 Y	1	BI	Recovery in speech and gait
[38]	China	Allogeneic	OEC & NPC	1 × 10^6^ & 2 × 10^6^	IC	5 Y	1	BI	Recovery in motor function
[44]	USA	Xenogeneic	Fetal Porcine cell	2 × 10^7^	IC	1.5–10 Y	5	BI, RS, NIHSS	Slight recovery, but 2 patients exhibited adverse events (seizure and motor deficit)
[38]	China	Allogeneic	OEC & NPC	1 × 10^6^ & 2 × 10^6^	IC & IT (NPC)	1–20 Y	4	BI	Recovery in gait
[36]	China	Allogeneic	UCBC & NPC	3 × 10^7^ (UC: IV), 1.5 × 10^7^ (UC: IT), 1.8 × 10^7^ (NPC: IT)	IV & IT	10 M & 2 Y	2	NIHSS, BI, mRS	Showed some degree of neurological recovery
